# Reduced cellular glucose transport confers natural protection against dextrose‐induced superoxide generation and endoplasmic reticulum stress in domestic hen

**DOI:** 10.14814/phy2.14816

**Published:** 2021-04-04

**Authors:** Arshag D. Mooradian, Michael J. Haas

**Affiliations:** ^1^ Division of Endocrinology, Diabetes, and Metabolism Department of Medicine University of Florida Jacksonville College of Medicine Jacksonville FL USA

**Keywords:** chicken aortic endothelial cells, endoplasmic reticulum stress, glucose transport, human coronary artery endothelial cell, oxidative stress

## Abstract

Normal blood glucose levels in avian species are two to fourfold higher than that in humans and the higher blood glucose levels in birds do not cause adverse effects. Endothelial cells isolated from the aorta of the domestic hen (*Gallus gallus* domesticus) and chicken aortic smooth muscle cells (CAOSMC) were compared to human coronary artery endothelial cells (HCAEC) and human primary aortic smooth muscle cells (HASMC). Superoxide (SO) generation was measured using a superoxide‐reactive probe. ER stress was measured using the placental alkaline phosphatase assay (ES‐TRAP). Glucose transport kinetics were determined using the ^3^H‐2‐deoxyglucose tracer. Dextrose‐induced SO generation and ER stress were significantly blunted in avian endothelial cells compared to human cells. The Vmax of glucose uptake (in nmoles/mg protein/min) in avian endothelial cells (0.0018 ± 0.0001) and smooth muscle cells (0.0015 ± 0.0007) was approximately 18–25 fold lower compared to the Vmax in HCAEC (0.033 ± 0.0025) and HASMC (0.038 ± 0.004) (all *p* < 0.0001). The Michaelis–Menten constant (Km) of transport was also significantly different (*p* < 0.0001) in avian species. The relative resistance of avian cells to dextrose‐induced oxidative stress and ER stress is mostly the result of reduced cellular dextrose transport.

## Graphical abstract

Normal blood glucose levels in avian species are twofold to fourfold higher than that in humans and birds do not suffer from diabetes‐related complications. Compared to human cells, dextrose‐induced oxidative and endoplasmic reticulum stress are blunted in avian coronary artery endothelial and smooth muscle cells. The reduced dextrose‐induced cell stress in avian cells is secondary to reduced cellular glucose uptake. Intrinsically reduced glucose transport in avian species protects birds from glucotoxicity.

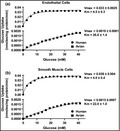

## 1 INTRODUCTION

Despite the advances made in the management of diabetes, a large number of people cannot achieve optimal blood glucose control and continue to develop vascular and neurologic complications. Although the main goal of therapy is to control blood glucose level along with managing the other known risk factors for vascular disease, circumstantial evidence suggests that therapeutic targeting of cellular stress may prevent diabetic complications, most notably the atherosclerotic vascular disease (Mooradian, 2016, 2017).

Birds are a unique model to examine potential strategies for preventing the complications of diabetes (Szwergold and Miller, 2014). The normal plasma glucose concentration of avian species is approximately 2–4 times of other species. Despite the high plasma glucose concentrations and documented insulin resistance (Braun & Sweazea, 2008; Sweazea & Braun, 2005), birds do not develop any known complications of diabetes, and they outlive mammals of comparable body size (Braun & Sweazea, 2008; Holmes et al., 2001; Speakman, 2005). This natural resistance to hyperglycemia‐related tissue damage has been attributed partly to reduced protein glycation (Zuck et al., 2017), higher levels of carbonyl‐scavenging amino acids in plasma (Szwergold and Miller, 2014), and lack of the gene for the receptor for advanced glycation endproducts (RAGE) (Sessa et al., 2014). However, the absence of RAGE in birds has yet to be confirmed at the protein level and species‐specific differences in defenses against oxidative stress may also be important for the ability of birds to live with chronic relative hyperglycemia (Klandorf et al., 2001).

Oxidative stress and endoplasmic reticulum (ER) stress are implicated in the pathogenesis of complications of diabetes (Mooradian, 2016; Mooradian & Haas, 2011). In the present communication, we report that the relative resistance of birds to the ravages of hyperglycemia may be related to their natural resistance to dextrose‐induced oxidative and ER stress and this resistance can be attributed to decreased glucose transport in the avian cells.

## 2 MATERIALS AND METHODS

### 2.1 Materials

Lipofectamine was purchased from Invitrogen. Kits for the preparation of plasmid DNA were purchased from Qiagen. Chicken aortic smooth muscle cells (CAOSMC), growth medium, and subculturing supplies were purchased from Cell Applications, Inc. Human primary aortic smooth muscle cells (HASMC), human primary coronary artery endothelial cells (HCAEC), growth medium, Dulbecco's phosphate‐buffered saline, penicillin/streptomycin/amphotericin B, Hank's Balanced Salt Solution (HBSS), and subculturing supplies were purchased from ATCC. ^3^H‐2‐deoxyglucose was purchased from Perkin Elmer. The chemiluminescent secreted alkaline phosphatase (SAP) substrate disodium 3‐(4‐methoxyspiro {1,2‐dioxetane‐3,2'‐(5'‐chloro)tricyclo [3.3.1.13,7]decan}‐4‐yl)phenyl phosphate (CSPD) was purchased from CloneTech. The superoxide anion probe 2‐methyl‐6‐(4‐methoxyphenyl)‐3,7‐dihydroimidazo[1,2‐A]pyrazin‐3‐one hydrochloride (MCLA) was purchased from Invitrogen. Heat‐inactivated fetal bovine serum (FBS) was purchased from Hyclone. Minimum essential medium (Eagle) (MEM) was purchased from Lonza. Collagenase and dispase were purchased from Worthington Biochemical Corporation. All other reagents were purchased from either Sigma Chemical Company or Thermo‐Fisher Scientific.

### 2.2 Cell culture

Avian artery endothelial cells were prepared as previously described (Campen & Davis, 1993). The experiments were conducted according to established animal welfare guidelines and the protocol for isolating endothelial cells for the aorta of chickens was approved by the animal use committee at the University of Florida.

Briefly, six‐week‐old hens (n = 6) were euthanized and their aortas were aseptically removed. The aortas were placed in a petri dish containing phosphate‐buffered saline (137 mM NaCl, 2.7 mM KCl, 10 mM Na_2_HPO_4_, 1.8 mM KH_2_PO_4_) (PBS) at 37°C and connective tissue and blood clots were removed. The aortas were then sliced lengthwise and pinned down in a paraffin‐filled petri dish and 8 ml of collagenase/dispase solution was poured on top. The interior surface of the aorta was scraped with a scalpel blade (#10) every 10‐minutes for a total of 30‐minutes. The collagenase/dispase solution was transferred to a 15‐ml, sterile conical centrifuge tube containing 8‐ml of MEM/10% FBS and 500 U/ml heparin. The cells were collected by spinning the tube in a centrifuge at 1,000 *g* for 10‐minutes at 25°C, and the supernatant was discarded. The pellet was suspended in 10‐ml of MEM/10% FBS and passed through a 210 µm nylon mesh. The filtrate was then passed through a 30 µm nylon mesh and the cell aggregates were placed in tissue culture plates in MEM containing 10% FBS, 100 U/ml penicillin, 100 µg/ml streptomycin, 2 mM l‐glutamine, and 0.1% Fungizone. These cells were Von Willebrand factor positive and smooth muscle alpha‐actin negative.

CAOSMC were cultured in Chicken Smooth Muscle Cell Growth Medium supplemented with a proprietary mixture of fetal chicken serum, growth factors, and antibiotics. These cells were positive for smooth muscle‐specific alpha‐actin antigen expression.

HASMC were cultured in Vascular Cell Basal Medium containing 5 ng/ml recombinant human basic fibroblast growth factor, 5 µg/ml insulin, 50 µg/ml ascorbic acid, 10 mM l‐glutamine, 5 ng/ml recombinant human epidermal growth factor, 5% FBS, 10 U/ml penicillin, 10 µg/ml streptomycin, and 25 ng/ml amphotericin B. These cells were factor VIII negative and smooth muscle alpha‐actin positive.

HCAEC were cultured in Vascular Cell Basal Medium supplemented with 5 ng/ml recombinant human epidermal growth factor, 0.2% bovine brain extract, 1 µg/ml hydrocortisone hemisuccinate, 0.5 U/ml heparin sulfate, 50 µg/ml ascorbic acid, 10 mM l‐glutamine, 2% FBS, 10 U/ml penicillin, and 10 µg/ml streptomycin. All cells were plated and subcultured as described by the supplier and maintained in a humidified incubator in 5% CO_2_ at 37°C. These cells were Von Willebrand factor positive and smooth muscle alpha‐actin negative.

All cell cultures were free of mycoplasma tested with the direct agar method (Hayflick, 1965).

### 2.3 Measurement of superoxide generation

Superoxide (SO) generation was measured using MCLA chemiluminescence as previously described (Sheikh‐Ali et al., 2010). Cells were treated with various concentrations of d‐glucose and MCLA was added to a final concentration of 1 µmol/L in Hank's balanced salt solution (HBSS) containing 1.26 mM CaCl_2_, 5.37 mM KCl, 0.44 mM KH_2_PO_4_, 0.49 mM MgCl_2_·6 H_2_O, 0.41 mM MgSO_4_·7 H_2_O, 136.7 mM NaCl, 4.2 mM NaHCO_3_, 0.34 mM Na_2_HPO_4_, and 5.5 mM d‐glucose. Superoxide‐induced chemiluminescence was measured with a luminometer and is expressed in relative light units (RLU).

### 2.4 Measurement of ER stress

ER stress was measured using the ES‐TRAP assay as previously described (Hiramatsu et al., 2006). The cells in six‐well plates were transfected with 2 µg of the plasmid pSEAP2.control using 5 µl of Lipofectamine and 24‐hours later the cells were treated as described in each figure. After 24‐hours, the conditioned medium was collected and SAP activity was assessed as follows. Twenty‐five microliters of conditioned medium was heated at 55°C for 30‐minutes to inhibit endogenous alkaline phosphatase activity. SAP reaction buffer was added to each sample (75 µl) as well as the chemiluminescent substrate CSPD and the samples were incubated at room temperature for 30‐minutes. Chemiluminescence was measured on a Turner Biosystems luminometer (10 s for each sample). Protein was isolated from the remaining cells after washing with HBSS three times, at which time 200 µl of sample buffer (2% sodium dodecyl sulfate (SDS), 10% glycerol, 60 mM tris(hydroxymethyl)aminomethane hydrochloride (Tris‐Cl) (pH 6.8), 1x HALT protease/phosphatase inhibitor cocktail) was added. The lysed cells were scraped from the plate and transferred to 1.5‐ml microcentrifuge tubes, sonicated briefly to reduce viscosity, at which time they were subjected to centrifugation at 12,000 *g* for 10‐minutes at 4°C. The protein content in each cell lysate, as well as the conditioned medium, was measured using the bicinchoninic acid (BCA) assay (Smith et al., 1985).

### 2.5 Glucose uptake measurement

Cells in 24‐well plates were grown to 90% confluence before measuring uptake of [^3^H]‐2‐deoxyglucose in the presence of increasing amounts of unlabeled dextrose as previously described (Ueyama et al., 1999). Glucose uptake was measured at 20 min, as preparatory studies demonstrated to be within the linear range of the assay. Radioactivity was measured with a Packard Tri‐Carb liquid scintillation counter (PerkinElmer, Inc.) and glucose uptake was normalized to protein content, which was measured in each well using the BCA assay. The kinetic constants of the facilitated transport were estimated by stepwise non‐linear regression analysis (Graph Pad Prism version 8.0; Graph Pad Software) performed to estimate the maximal glucose transport rate (Vmax) and glucose concentration at half‐maximal transport (Km: Michaelis‐Menten constant).

### 2.6 Statistical analysis

All results are expressed as mean ±S.D. Analysis of variance (ANOVA) followed by the Neuman–Keuls procedure for subgroup analysis was carried out using Statistica. Significance was defined as a two‐tailed *p* < 0.05.

## 3 RESULTS

### 3.1 The effect of dextrose on SO generation and ER stress in endothelial cells

**FIGURE 1:**
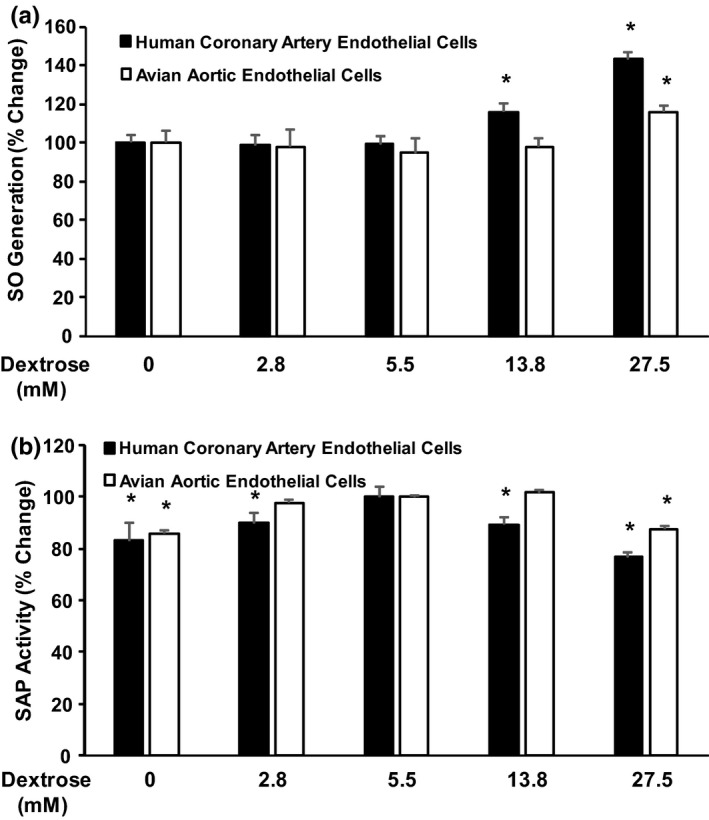
The effect of varying concentrations of dextrose on (a) superoxide (SO) generation and (b) endoplasmic reticulum (ER) stress in human coronary artery endothelial cells (HCAEC) and avian aortic endothelial cells. ER stress is the reverse of secreted alkaline phosphatase (SAP) activity. (N = 6 cell cultures for each figure) *, *p* < 0.05 relative to cells exposed to 5.5 mM dextrose. All results are expressed as mean ± S.D. Analysis of variance (ANOVA) followed by the Neuman–Keuls procedure for subgroup analysis was carried out using Statistica

The SO generation was measured one hour after treating the cells with varying concentrations of dextrose (0–27.5 mM). In avian aortic endothelial cells (Figure 1a, clear bars), SO generation was consistent and did not change in cells exposed to 0, 2.8, 5.5, and 13.8 mM dextrose, however in cells treated with 27.5 mM dextrose, SO generation increased by 15.8% (*p* < 0.005). In contrast, SO generation in HCAEC (Figure 1a, black bars) increased in cells treated with 13.8 and 27.5 mM dextrose (16.2% and 43.0%, respectively; *p* < 0.01 and *p* < 0.0003, respectively). The effect of dextrose on ER stress is shown in Figure 1b. The increased ER stress is indicated by the reduction in SAP activity in the culture medium. In avian aortic endothelial cells (Figure 1b, clear bars), SAP activity was lower only with exposure to 0 and 27.5 mM dextrose (*p* < 0.04 and *p* < 0.02, respectively). In contrast, in HCAEC (Figure 1b, black bars), SAP activity decreased in cells exposed to 0, 2.8, 13.8, and 27.5 mM dextrose relative to cells exposed to 5.5 mM dextrose (*p* < 0.001, *p* < 0.002, *p* < 0.001, and *p* < 0.0009, respectively).

### 3.2 Glucose uptake in human and avian endothelial and smooth muscle cells

**FIGURE 2:**
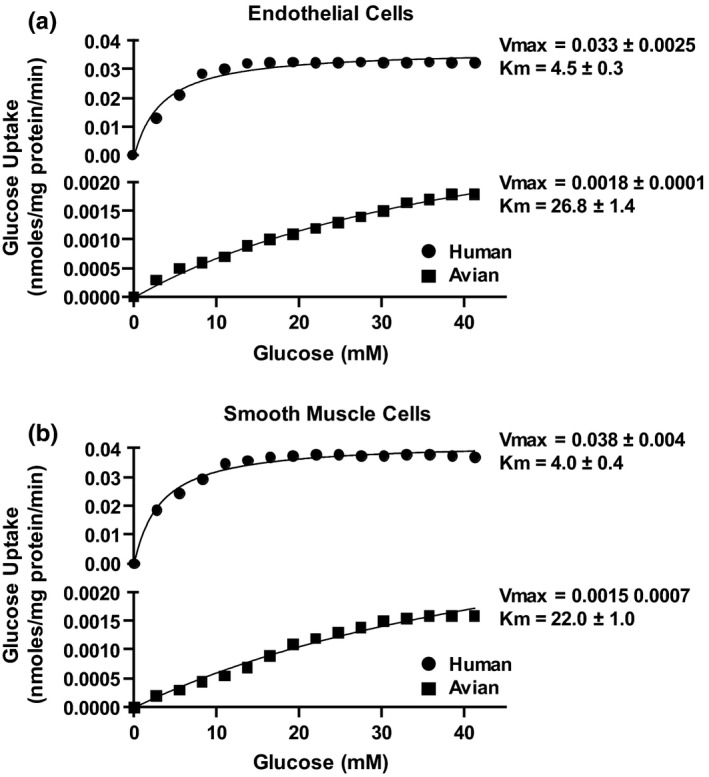
Kinetics of glucose uptake in human and avian endothelial (a) and smooth muscle cells (b). Glucose uptake was measured and Vmax (nmoles/mg protein/min) and Km (mM) were determined. Endothelial cells were obtained from human coronary arteries and avian aortae. Smooth muscle cells, purchased from ATCC, were obtained from human and avian coronary arteries and avian aortae, respectively. Vmax = maximal velocity of cellular glucose uptake; Km = Michaelis‐Menten constant of glucose transport. Glucose uptake was significantly more efficient in human endothelial and smooth muscle cells relative to avian endothelial and smooth muscle cells (*p* < 0.05), (N = 6 cell cultures for each data point). All results are expressed as mean ± S.D. Analysis of variance (ANOVA) followed by the Neuman–Keuls procedure for subgroup analysis was carried out using Statistica

Treatment with cytochalasin B reduced glucose uptake by greater than 95% in all cell lines (data not shown). The maximal glucose transport rate (Vmax in nmoles/mg protein/min) in HCAEC was 0.033 ± 0.0025 compared to 0.0018 ± 0.0001 in avian aortic endothelial cells (*p* < 0.0001) (Figure 2). The Km of transport (glucose concentration at half‐maximal transport) was significantly higher in avian aortic endothelial cells (26.8 ± 1.4 mM) compared to HCAEC (4.5 ± 0.3 mM) (*p* < 0.0001). Similarly, the Vmax of glucose uptake was greater in HASMC (0.038 ± 0.004) than in CAOSMC (0.0015 ± 0.0007) (*p* < 0.0001). Likewise, Km values for HASMC were significantly lower than in CAOSMC (4.0 ± 0.4 mM vs. 22.0 ± 1.0 mM, respectively) (*p* < 0.0001).

## 4 DISCUSSION

The principal aim of these studies was to understand why blood glucose concentrations that cause toxic tissue injury in humans do not cause the same toxicity in avian species. The results of the present study show that dextrose‐induced superoxide generation and ER stress are significantly reduced in endothelial cells derived from the aorta of chickens compared to the changes observed in human coronary artery endothelial cells. The SO generation in chicken cells increased with 27.5 mM dextrose (the high end of normal chicken blood glucose levels) compared to the cells treated with lower dextrose concentrations. This increase was significantly less than the SO generation in human cells treated with 27.5 mM dextrose (human hyperglycemic concentration) (Figure 1a). The ER stress as measured by the decrease of SAP activity occurred in hypoglycemic and hyperglycemic conditions in human cells. This has been previously observed in previously published manuscripts (Mooradian et al., 2016). A similar profile is observed in chicken cells albeit with reduced sensitivity as the SAP decrease was observed only in the extreme spectrum of low (0 mM) and the higher end of normal chicken dextrose concentrations (27.5 mM) (Figure 1b). Since both oxidative stress and ER stress are implicated in the pathogenesis of complications of diabetes (Mooradian, 2016; Mooradian & Haas, 2011), it is possible that the relative immunity of birds to hyperglycemia‐related complications is partly the result of their reduced cellular stresses. It is noteworthy that ER stress was measured only with ES‐TRAP assay and the other cellular markers of ER stress such as the biochemical changes observed in unfolded protein response was not measured as the biochemical tools used in these measurements have not been validated for avian species.

The relative protection against hyperglycemia‐related SO generation and ER stress in avian cells compared to human cells is mostly attributable to significantly reduced cellular glucose transport in avian cells (Figure 2). Measurements of glucose transport kinetics in both endothelial cells and smooth muscle cells revealed significantly lower Vmax and higher Km of glucose transport in chicken cells as compared to human cells (Figure 2). Whether these differences are intrinsic to avian biology or are a secondary adaptation to chronic hyperglycemia is not known. However, the latter postulate is less likely to be true as these cells are maintained in culture media containing only 5.5 mM dextrose and any adaptive change would have likely been reversed following several cell culture passages. In addition, microarray studies have shown that glucose transporter expression in chicken tissues is different from that in mammals (Byers et al., 2017; Kono et al., 2005). There are thirteen members of mammalian glucose transporters (GLUTs 1–12 and H^+^‐myo‐inositol transporter) and humans have a duplicated GLUT member, GLUT 14. However, there are no chicken orthologs of mammalian GLUT 4 and GLUT 7 and some chicken GLUT members do not have corresponding orthologs in mammals (Byers et al., 2017). In humans, GLUT 4 is an insulin‐regulated glucose transporter that is responsible for glucose uptake into fat and muscle cells, while. GLUT 7 is a hexose (glucose/fructose) transporter primarily expressed in the small intestine and colon. Thus, chickens intrinsically lack insulin‐responsive transporter GLUT 4, while insulin non‐responsive GLUT 1 mRNA is expressed in most tissues with the highest levels in the brain and adipose tissue, GLUT 2 is expressed only in the liver and kidney, GLUT 3 is highly expressed in the brain and GLUT 8 is expressed ubiquitously (Byers et al., 2017; Kono et al., 2005). The precise role of these glucose transporters in avian species is not known.

One of the limitations in this study is that the experiments are done on commercial breeds of chickens rather than avian species in the wild. Nevertheless, the overall evidence suggests that intrinsically reduced glucose transport in avian species protects birds from glucotoxicity. It remains to be seen if endothelial glucose transport could be a therapeutic target and if downregulating glucose transport into endothelial cells would prevent accelerated atherosclerosis in people with diabetes.

## CONFLICT OF INTEREST

The authors have no conflicts of interest to declare.

## AUTHOR CONTRIBUTION

Arshag D. Mooradian: Conceptualization, Methodology, Formal Analysis, Writing—Original Draft, Writing Review and Editing. Michael J. Haas: Conceptualization, Methodology, Formal Analysis, Writing—Original Draft, Writing Review and Editing.
